# Evaluation of the immune responses induced by four targeted DNA vaccines encoding the juvenile liver fluke antigen, cathepsin B in a mouse model

**DOI:** 10.1186/1479-0556-10-7

**Published:** 2012-08-31

**Authors:** Rama Jayaraj, David Piedrafita, Terry Spithill, Peter Smooker

**Affiliations:** 1 Biotechnology & Environmental Biology, School of Applied Sciences, RMIT University, Bundoora West Campus, PO Box 71, Bundoora, Vic 3083, Australia; 2 Allied Health Program, School of Environmental and Life Science, Charles Darwin University, Casuarina, NT 0810, Australia; 3 Animal Biotechnology Research Laboratories, Physiology Department, School of Biomedical Sciences, Monash University, Clayton, Vic, 3800, Australia; 4 Department of Agricultural Science, Latrobe University, Melbourne, Vic, 3086, Australia; 5 Biotechnology & Environmental Biology, School of Applied Sciences, RMIT University, Bundoora West Campus, PO Box 71, Bundoora, Vic, 3083, Australia; 6 School of Environmental and Life Sciences, Charles Darwin University, Casuarina, NT, 0909, Australia

**Keywords:** Cathepsin B2, *Fasciola hepatica*, DNA vaccination, CTLA-4, MCP3

## Abstract

**Background:**

Liver fluke can infect cattle and sheep, and is also emerging as a human pathogen in developing countries. Cathepsin B (Cat B2) is a major cysteine protease secreted by the juvenile flukes. To enhance the immune responses of Cat B2, the cDNA sequence was fused with four different DNA vaccine vectors. The induced cellular and antibody responses were compared in vaccinated mice.

**Methods:**

The following recombinant DNA vaccine constructs were constructed: empty vector VR1012 as negative control, cytoplasmic construct pVR1012 Cat B2, secretory construct pVR1020 Cat B2, chemokine-fused construct pMCP3 Cat B2 and lymph node targeting construct pCTLA-4 Cat B2. Plasmids were constructed using standard procedures, and positive constructs screened and selected using restriction digestion analysis followed by sequence analysis. The constructs were then tested in Cos-7 cells for *in vitro* expression, which was analysed using immunoblotting. Subsequently, female BALB/c mice were immunised with DNA constructs as vaccines. Elicited antibody responses were measured using ELISA. The ratio between IgG1 and IgG2a antibody responses was estimated among different vaccine groups. IgG antibody avidity assay was performed and the relative avidity index was calculated. The induced cytokine production from splenocytes of vaccinated animals was estimated using ELISPOT.

**Results:**

DNA vaccine constructs carrying Cat B2 were expressed in Cos-7 cell lines and encoded protein was recognised using western blotting using rat anti- cathepsin B antibody. DNA vaccines elicited high Cat B2- specific IgG, IgG1, IgE and also modest IgG2a antibody responses. Cat B2 specific IL-4 T cell responses were also observed in Cat B2 vaccinated mice. The comparison of immunogenic potential in each of these constructs was demonstrated as enhanced antibody responses on the lymph-node targeting vector pCTLA-4 Cat B2, the high antibody avidity of chemo-attractant pMCP3 Cat B2 and stronger T cellular responses of non-secretory DNA vaccine pVR1012 Cat B2 in vaccinated animals.

**Conclusion:**

This study showed that the targeting DNA vaccine strategies enhanced specific immune responses to juvenile fluke Cat B2. The results of our current study have demonstrated that a gene-based vaccine as an immunotherapeutic approach to combat *Fasciola* infection may be feasible.

## Background

The liver flukes *Fasciola hepatica* and *F .gigantica* cause an estimated 3.2 billion annual economic loss to the global agricultural community [1]. Liver fluke disease (fasciolosis) is an emerging human affliction, with an estimated 2–4 million people infected and a further 16 million people at risk of infection [1]. Due to the emergence of drug resistant strains and avoidance of chemical residues in animal food products such as milk and meat, vaccines are proposed as an alternative to current chemotherapy for fasciolosis [1]. Juvenile and immature stages of *F. hepatica* produce excretory and secretory (ES) material and cathepsin B (Cat B) is found to be a major part of the ES material [2,3]. The major action of Cat B is found to be assisting in the excystment and penetration of young flukes into intestinal peritoneal and hepatic tissues of the host [3,4]. Cathepsin proteases are purified from the ES products of immature *F. hepatica*, but the production of sufficient quantities of pure cathepsin is a time consuming and complicated. Certainly large quantities from tiny immature fluke are not possible. To solve this issue, recombinant cathepsin expression is ideal choice for vaccine studies. In our previous study, Cat B2 was evaluated as a recombinant protein vaccine, and shown to induce protective immune responses in rats [5]. The key role of Cat B in the biology of flukes has been demonstrated by its enzyme characteristics, RNA interference (RNAi) and vaccination studies, reviewed by Smooker and colleagues [6]. Therefore, Cat B is proposed to be a potential vaccine candidate against fasciolosis.

DNA vaccinations have been progressively used as a more attractive vaccine approach because they are capable of directly transfecting dendritic cells, and can stimulate both humoral and cellular immunity [7]. However, the generated specific antibody titres of DNA vaccination are generally observed to be far less than those induced by protein vaccination [8]. DNA vaccinations with *Fasciola* antigens have shown effectiveness of such vaccines in evoking immune responses [9-15]. In order to increase the antibody responses of DNA vaccines encoding antigen, a number of strategies have been employed. The most popular strategies applied are secretory and vaccine cytoplasmic vectors. For example, secretory and cytoplasmic DNA encoding *F. hepatica* glutathione S transferase, fatty acid binding protein and cathepsin L5 was investigated in mouse trials, and the encoded antigens evoked higher immune responses in the secreted form [14,16].

Another strategy includes the use of chemokines that improves the immunogenicity of poorly immunogenic antigens by targeting them to antigen presenting cells (APCs) via chemokine receptors [17]. MCP3 has been evaluated by virology researchers as a chemo-attractant of leukocytes [17]. Vaccination with constructs encoding CTLA-4 fusion proteins (which bind to CD80/86 of APC’s) can induce strong antibody responses and provides a novel generic DNA vaccine for the development of therapies against a wide range of diseases [18-23]. Targeting of APCs by CTLA-4 encoding ovalbumin was performed in pigs via gene gun delivery. This DNA vaccination induced an elevated antigen specific IgG, IgA, IgG1 and IgG2 antibody responses in pigs [24]. CTLA-4 mediated targeting and CpG motifs enhance immunogenicity in a DNA prime/protein boost strategy in sheep using *Fasciola* antigens [10].

The determination of specific IgG avidity in sera is generally useful in parasitic and viral infections including *Fasciola*[25], *Trypanosoma cruzi*[26], Rubella [27] and Mumps [26] for differentiating the acute and chronic stage of infection. The estimation of antibody avidity has also been widely used for analysing the vaccine efficacy of infectious diseases, where the stimulation of high avidity antibodies is required [28-30]. The avidity index (AI) is currently measured in vaccinated sera by ELISA with one more extra step: disassociation of the antigen-antibody complex with denaturing agents such as urea or thiocynate [27,31,32].

In order to assess the immunogenic property of an early and infective stage fluke secreting cysteine protease as a vaccine candidate, the humoral and cellular immune responses to various DNA vaccines encoding F. hepatica cathepsin B protease were investigated. This panel of constructs was analysed for in vitro expression with COS-7 cells and in vivo with BALB/c mice via the intramuscular route.

## Methods

### Construction and purification of DNA vaccines encoding Cat B2

Cloning of Cat B2 and the construction of DNA vaccines was previously performed [2]. The coding region for pro-cathepsin B2 was inserted into the DNA vaccines. The constructs to be tested were as follows: secretion of Cat B2 using the native signal peptide (pVR1012 Cat B2), secretion of Cat B2 using the TPA signal peptide (pVR1020 Cat B2), secretion of Cat B2 fused to murine MCP3 (pMCP3 Cat B2) and secretion of Cat B fused to murine CTLA4 (pCTLA-4 Cat B2). The DNA constructs were purified from one litre of *E. coli* BL21 DE3 pLysS (Novagen, USA) culture using an endotoxin free plasmid Giga kit (Qiagen Australia). The purified DNA was diluted in endotoxin free 0.9% saline solution at a concentration of 1 mg/ml.

### COS-7 cell expression of Cat B2

The panel of constructs were examined for protein secretion from COS-7 cells to confirm the functional expression of antigens. The expression and purification of Cat B2 from *S. cerevisiae* BJ 3505 cells proceeded according to Law *et al*. [2]. *In vitro* expression of Cat B2 was evaluated by transfecting COS-7 cells (a kind gift from Kemperley Dynon, Melbourne University, Parkville) using the lipofectamine^Tm^ LTX reagent (Invitrogen Australia). For each transfection, 4 μg of plasmid was added to 100 μL of DMEM medium without newborn calf serum (NCS) (Sigma-Aldrich Pty Ltd, USA) and 20 μL of lipofectamine LTX reagent. The mixture was incubated for 5 minutes at room temperature and then added to COS-7 cells. After incubation at 37°C for 24 hours, one mL of complex DMEM medium with NCS (10%) was added. After incubating for a further 48 hours at 37°C, the cells were washed with PBS and growth media without NCS and grown for a further 24 hours. After harvesting of COS-7 cells, the supernatant was concentrated using an Amicon ultra filtration unit and 20 μL of highly concentrated supernatant and/or yeast expressed Cat B2 was used for western blotting. Western blots were probed with rat anti-cathepsin B antibodies (1:100) and followed by anti- rat alkaline phosphatase (Invitrogen Australia) conjugated secondary antibodies (1:100) and reactive antibodies were visualised BCIP/NPT (Roche Diagnostics, Australia).

### Cathepsin B2 protein expression

*Fasciola hepatica* is the source of yeast expressed cathepsin B2. Expression and purification of cathepsin B from S. cerevisiae BJ 3505 cells proceeded according to Law et al. [2]. pFLAG cathepsin B2 transformants were grown at 28°C using shaking (120 rpm) in 10 mL minimal medium. After 72 hours growth, the cells were centrifuged and the cell pellet was put into YPHSM medium (one litre) and incubated with shaking at 120 rpm for 72 hours.

### Immunization

Ethics approval to perform DNA vaccination in BALB/c mice was obtained from RMIT University Animal Ethics Committee, Melbourne, Australia. Groups of five 6–8 week old BALB/c female mice were immunised with VR1012 as the control, VR1012 Cat B2, VR1020 Cat B2, MCP3 Cat B2 and CTLA-4 Cat B2 as vaccines. The plasmid DNA was administrated three times at two week intervals via an intramuscular injection to the thigh region. Mice received 100 μg of DNA in 100 μL of 0.9% endotoxin free saline solution (50 μL each thigh) on weeks 0, 2, 4. Mice were bled and sera were sampled on weeks 4, 6, 8 and 10.

### ELISA

The sterile ELISA plates (96 well) were coated with yeast expressed Cat B2 at 5 μg/ mL in carbonate bicarbonate buffer pH 9.6 and incubated overnight at 4°C. After blocking, sera from individual mice were serially diluted (1:100), loaded onto the plates and incubated at 37°C for 2 hours under gentle shaking. The bound antibodies were detected using anti-mouse HRP conjugated IgG antibody ((Sigma-Aldrich Pty Ltd, USA) (1:3000 dilution)), followed by the addition of 3’, 3’, 5’, 5’-tetramethylbenzidine (TMB substrate, BD Pharmingen, USA). The reaction was stopped by adding 2 M sulphuric acid. Reciprocal titres were calculated as the dilution that yielded an OD_450_ absorbance of 0.2.

For detection of antibody isotypes at week 10, biotin conjugated rat anti-mouse IgE, IgG1 and IgG2a (BD Pharmingen, USA) was added at 1:500 dilution and incubated for one hour, followed by washing and the addition of peroxidase-conjugated goat anti-rat IgG (at 1:500 dilution). The absorbance read at 450 nm on an ELISA reader. The ratio between IgG1 and IgG2a antibody responses was also estimated to compare the Th1/Th2 ratio among different vaccine groups.

### Antibody avidity assay

An antibody avidity assay was performed as described elsewhere [29,30] with the following modifications. Yeast expressed Cat B2 (5 μg/mL) was used to coat 96 well plates. The sera collected from individual mice were added to all wells according to their antibody titre value (OD450 absorbance of 0.2) and incubated for one hour at 37°C, followed by the addition of an increasing concentration of urea to 0, 1, 2, 3, 4, 5, 6 and 7 M and further incubation for 30 minutes at 37°C. The humoral responses were assessed using anti-mouse HRP conjugate (Sigma-Aldrich Pty Ltd, USA) and developed as described for the ELISA above. The relative avidity index was calculated as the urea concentration required to reduce the binding percentage to 50%.

### Elispot assay

Vaccinated mice were sacrificed at week 10. Spleens were extracted from two animals in each group, crushed, cells washed two times using RPMI medium and incubated in ACK lysis buffer (0.15 M NH_4_Cl; 10 mM KHCO_3_; 0.1 Mm Na_2_EDTA, pH 7.4) for 5 minutes. Cells were washed in 1 mL RPMI medium and suspended at a concentration of 1x 10^6^ cells/90 μL. Methanol treated 96 well multi screen plates (Millipore) were coated with 100 μL of 5 μg/mL of anti-mouse interleukin-4 (IL-4) overnight, followed by washing with PBS-Tween 20_,_ blocking with 5% skim milk in PBS for 2 hours and a further washing step with PBS-Tween 20. Splenocytes (1X10^6^) were then added to each well. Splenocytes were stimulated with 250 μg/mL of cathepsin B or concanavalin A (Sigma-Aldrich Pty Ltd, USA). Cultures were incubated at 37°C in a 5% CO_2_ humidifier for 21 hours. Following washing with PBS, biotinylated rabbit polyclonal anti- IL-4 (BD Pharmingen, USA) in PBS was added to wells and incubated at room temperature for 2 hours. After washing, strepavidin-alkaline phosphatase (Sigma-Aldrich Pty Ltd, USA) was added and incubated at room temperature for 1 hour. After washing three times with PBS-Tween 20 and two washes with sterile Milli Q water, ELISA substrate solution were added and spots were counted using a dissection microscope. Experiments were performed in triplicate and results were expressed as the mean number of cytokine secreting cells per 10^6^ splenocytes.

### Western blot

For assessing the Cat B2 specific antibodies in pooled sera of vaccinated mice groups, Cat B2 was separated (SDS-PAGE) and transferred to nitrocellulose membranes. After blocking, the membrane was probed with antisera (1:100 dilution) from all vaccinated groups and then probed with anti-mouse alkaline phosphatase conjugated (1:2000 dilution) and then finally developed by BCIP/NPT. Antisera were self raised antibodies in rats vaccinated with Cat B2 (from our study, [33]).

### Statistical analysis

Mean and standard deviation was calculated for the analyses of antibody titre, antibody avidity and ELISPOT assays. The data were analysed using Graphpad Prism (3.02 software, San Diego, USA). The association between paired and continuous, normally distributed data were estimated using Wilcoxon test, whereas the Mann–Whitney U-test was used for non-normally distributed continuous data. Comparisons were considered to be significant at *P* values of < 0.05.

## Results

### DNA vaccine purification and COS-7 cell expression

**Figure 1:**
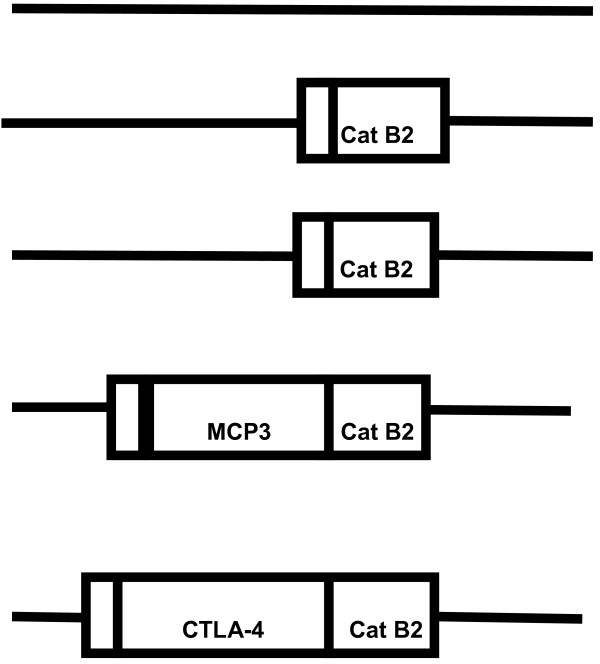
**Diagrammatic representation of the DNA vaccine constructs VR1012, VR1012 Cat B2, VR1020 Cat B2, MCP3 Cat B2 and CTLA-4 Cat B2.** The respective coding regions are detailed as rectangles (Cat B2) and signal peptide as small rectangles. VR1012 Cat B2 has the native Cat B2 signal peptide. VR1020 Cat B2 has the TPA signal peptide encoded in the vector. MCP3 Cat B2 and CTLA4 Cat B2 have their own signal peptides.

**Figure 2:**
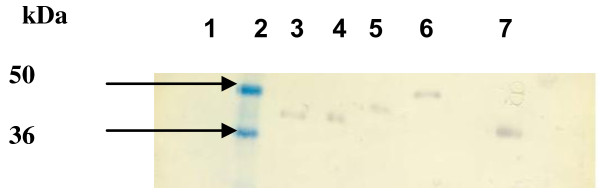
**Western blot detection of encoded proteins secreted from COS-7 cells transfected with DNA vaccine vectors.** Lane 1, VR1012 transfected cell supernatant; lane 2, pre-stained protein marker; lane 3, VR1012 Cat B2 cell supernatant; lane 4, VR1020 Cat B2 cell supernatant; lane 5, MCP3 Cat B2 cell supernatant; lane 6, CTLA4 Cat B2 cell supernatant; lane 7, yeast expressed Cat B as a positive control.

The constructs are depicted in Figure 1. Analysis of western blots probed with Cat B2 specific rat sera revealed the secretion of proteins from COS-7 cells in each construct (Figure 2). Bands corresponding to pro-Cat B2 were observed, and also the recognition of 36 kDa and 50 kDa fusion MCP3 or CTLA4- Cat B2 protein bands was observed. There was no reactivity observed when COS-7 cells were transfected with VR1012 as expected.

### Humoral immune responses

**Figure 3:**
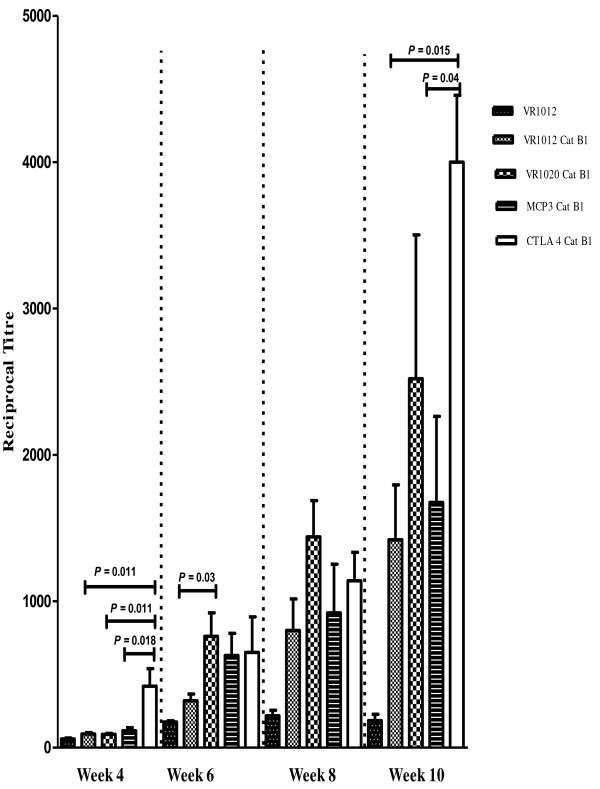
**Cat B2 specific IgG antibody responses in vaccinated mice at bi-weekly intervals from weeks 4 to 10 after the first vaccination.** At every time point, all vaccinated groups with Cat B2 encoding constructs showed enhanced antibody titres when compared with control vectors *P* < 0.05).

Cat B2 specific antibody titres were induced in BALB/c mice after vaccination with DNA vaccines (Figure 3). Mean IgG antibody titres generated in mice immunised with the CTLA-4-Cat B2 were significantly higher (*P* < 0.05) than all other constructs at week 4. At week 6, the mean antibody titre of VR1020 encoding Cat B2 vaccinated mice showed statistically significant higher than the mice vaccinated with VR1012 Cat B2. There was no significant difference between the mean titres of any of the remaining test groups at weeks 6 and 8. However, at week 10 the mean antibody titres in CTLA-4 tagged Cat B2 vaccinated group was again higher than the VR1012 Cat B2 and MCP3 Cat B2 groups (*P <* 0.01; *P <* 0.05).

**Figure 4:**
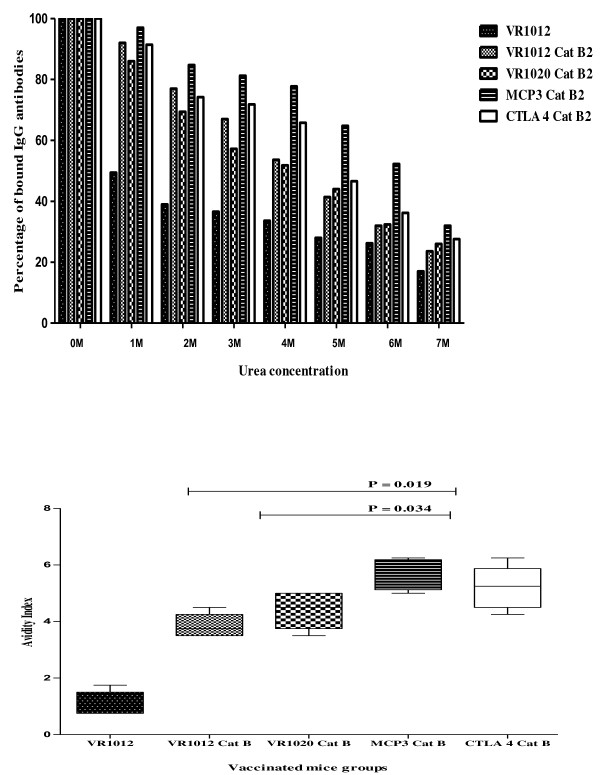
**(A). Measurement of the avidity of IgG antibody responses to Cat B2.** Values are normalised to 100 % at 0 M.; (**B**). Relative avidity index of anti-Cat B2 specific IgG antibodies in immunised mice.

The panel of DNA vaccines induced relatively high IgE and IgG1 responses, and modest IgG2a responses (Figure 4). All Cat B2 encoding DNA vaccines showed a statistically significant IgE antibody responses compared to the control vaccine (*P < 0.01*). The same trend was observed in specific IgG1 and IgG2a antibody responses to Cat B2 encoding DNA vaccines (*P <* 0.05).

### Avidity of IgG antibody responses

The rapid drop in antibody avidity observed for the VR1012 group (ie: the negative control) reflects the non-antigen specific nature of binding in this group (Figure 4). MCP3 Cat B2 vaccinated mice sera showed a higher percentage of binding to antigen as urea concentrations increased compared to other groups. This showed that a higher urea concentration would be required to disrupt Cat B2 /IgG interactions (> 7 M) in the mice vaccinated with MCP3 Cat B2. The relative avidity index of MCP3 Cat B2 vaccinated mice sera were significantly higher than VR1012 Cat B2 and VR1020 Cat B2 group (P < 0.05).

### Immunoblotting

**Figure 5:**
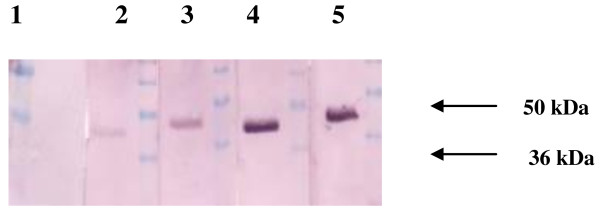
**Western blot of yeast expressed Cat B2 protein probed with pooled sera of vaccinated mice.** Sera from mice vaccinated with: Strip 1, VR1012; strip 2, VR1012 Cat B2; strip 3, VR1020 Cat B2; strip 4, MCP3 Cat B2, strip 5, CTLA4 Cat B2 sera. The individual strips contain See Blue pre-stained protein markers. Each individual immunoblot shows reactivity except the control DNA vaccinated sera.

Sera from all groups vaccinated with Cat B2 constructs were able to recognise the protein (Figure 5). No signal was detected for control vaccine sera. In these semi-quantitative blots, MCP3 and CTLA-4 construct showed stronger reactivity than other vaccinated groups.

### Cellular immune responses

**Figure 6:**
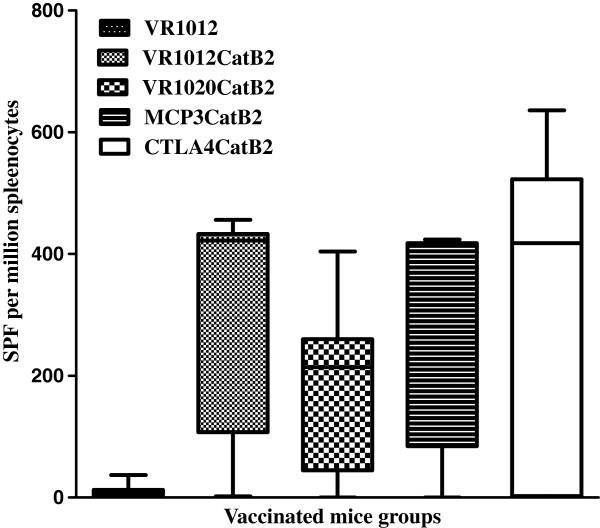
**Quantitative determination of IL-4 secreting spots (ELISPOTs) in vaccinated groups.** The mean and standard deviation are presented here. Each vaccine test group yielded significantly higher numbers of IL4 secreting cells than the control (*P* < 0.01) (SPF -spot forming T cells).

Vaccination with all plasmids encoding Cat B2 induced a highly significant increase in IL-4 cytokine secreting cells (*P <* 0.005) compared to the control groups. as illustrated in Figure 6. Interestingly, the native signal peptide carrying construct (VR1012 cat B2) vaccinated mice produced the highest level of IL-4 cytokine production. However, there was no significant difference between the numbers enumerated from any group vaccinated with a cathepsin B encoding construct.

## Discussion

This study demonstrates that delivery of the juvenile and immature *F. hepatica* antigen Cat B2 via DNA vaccine vectors induces humoral and cellular responses in BALB/c mice. Antigen availability for B cell priming is an essential factor in designing DNA vaccinations for the induction of humoral responses [34]. In DNA vaccination, small amounts of secreted protein would aid to select B cells with high avidity [8]. The COS-7 transfection analysis with the panel of DNA vaccine vectors indicated that *in vivo* expressed Cat B2 protein should be secreted from cells and be available for the priming of B cells.

There are a number of reports explaining the speed and magnitude of IgG antibody induction in mice vaccinated with secretory vaccine vectors [13,35-38]. A previous report by Smooker *et al*. (2001) showed that after DNA vaccination with constructs encoding liver fluke antigens, IgG antibody responses peaked (1/2000) at week 8 and remained high for 20 weeks. In this study, constructs encoding liver fluke FABP only induce antibodies when delivered in a form that will secrete FABP from the host cell. Antigen availability for B cell priming is an essential factor in designing DNA vaccinations for the induction of humoral responses [18].

DNA vaccination with the VR1012 DNA vaccine encoding *F. hepatica* cathepsin L showed total IgG antibody titre increased by week 8 and attained a peak at week 13 (1/2000) [16]. In a similar pattern, VR1012 encoding Cat B2 elicited IgG antibody responses that reached its peak antibody titre at week 10 in our study (1/1500).

The kinetics of antibody induction was different between the four constructs, with CTLA-4 Cat B2 inducing a strong response at the earliest time point measured (4 weeks) compared to the three other constructs. Therefore it appears that the CTLA-4 fusion construct generally induces titres faster than other constructs, and induced a high antibody titre. This confirms what has been seen in several studies [18,19] as one of the major advantages of using CTLA-4 fusion constructs is the early induction of immune responses [19].

In our experiments, the MCP3 construct did not induce very high antibody responses and other studies [38,39] have shown increased responses. The reason is unknown, and is presumably related to the specific combination of antigen and chemokine that is expressed. MCP3 is supposedly acting in a similar way to CTLA-4 in delivering antigen to antigen presenting cells, but not giving the expected increase. This confirms that CTLA-4 is the superior targeting system in these experiments.

In our study, Cat B2 DNA vaccines induced very modest IgG2a antibody response and dominant IgG1 and IgE antibody responses. The dominant IgE antibody response of cysteine proteases was observed in our previous study with *F hepatica* cathepsin L5 DNA vaccination in mice [16]. The presence of *Fasciola* specific IgE antibody and eosinophil responses is a good indicator of acquired immunity which has been demonstrated elsewhere [40-42]. In a rat trial with recombinant cathepsin L, vaccination induced significantly higher specific IgG1 antibodies in vaccinated groups than in the control group [43].

One way of characterising antibody responses is to estimate the avidity of antibodies. Our results clearly show the sharp drop in binding in the control group, which is obviously reflective of non-specifically bound antibody. Rainczuk *et al.*[30] tested the relative avidity of DNA vaccines where malarial antigen MSP4-5 was fused with MCP3 or CTLA-4 and found that the avidity of both these constructs were comparable. The relative avidity of MCP3 Cat B2 induced antibodies were higher than those induced by other vaccines, as inferred by a urea IgG ELISA. MCP3 has been shown to bind to chemokine receptors CCR1, CCR2, and CCR3 which are all expressed on immune cells.

Generally, Th1 and Th2-associated responses in the murine system are reflected by IgG2a and IgG1 isotypes respectively [44]. Dendritic cells are crucial for processing and presenting antigens to stimulate naїve T lymphocytes, and also differentiate into a Th1 or Th2 T cell responses which provide T cells with costimulatory signals, CD80/CD86 [45]. In a mouse study with experimental *Fasciola* infection, spleen cells from BALB/c exhibited a Th2 response, producing high levels of the cytokines IL-4 and IL-5, and low levels of IFN-gamma and IL-2. In contrast, C57BL/6 mice showed a mixed Th1/Th2 response. The induction of IL4 by fluke infection in mice has been well documented by many groups [46-49]. As stated above, the migratory juvenile and adult liver fluke ES material elicits Th2 responses [41,50].

## Conclusion

In summary, our results confirmed that the juvenile fluke antigen Cat B2 B can elicit cellular and humoral responses when delivered as a DNA vaccine in a murine model. Our results also indicated that exploring various fusions of DNA vaccination strategies may be an effective approach to further enhance the potency cathepsin B against challenge infection in animal models. We will plan to expand this study in future with target animals, cattle and sheep to see how the protection given by these vaccine.

## Competing interests

The authors declare that they have no competing interest.

## Authors’ contribution

JR conceived of the study, participated in its design and coordination, carried out the review, and drafted the manuscript. PD and ST conceived of the study, participated in its design and helped draft the manuscript. PS conceived of the study, participated in its design and helped draft the manuscript. All authors read and approved the final manuscript.
